# A phylogenomics approach for selecting robust sets of phylogenetic markers

**DOI:** 10.1093/nar/gku071

**Published:** 2014-01-28

**Authors:** Salvador Capella-Gutierrez, Frank Kauff, Toni Gabaldón

**Affiliations:** ^1^Bioinformatics and Genomics Programme. Centre for Genomic Regulation (CRG) and UPF. Doctor Aiguader, 88. 08003 Barcelona, Spain, ^2^Universitat Pompeu Fabra (UPF). 08003 Barcelona, Spain, ^3^University of Kaiserslautern, Molecular Phylogenetics, Postfach 3049, 67653 Kaiserslautern, Germany and ^4^Institució Catalana de Recerca i Estudis Avançats (ICREA), Pg. Lluís Companys 23, 08010 Barcelona, Spain

## Abstract

Reconstructing the evolutionary relationships of species is a major goal in biology. Despite the increasing number of completely sequenced genomes, a large number of phylogenetic projects rely on targeted sequencing and analysis of a relatively small sample of marker genes. The selection of these phylogenetic markers should ideally be based on accurate predictions of their combined, rather than individual, potential to accurately resolve the phylogeny of interest. Here we present and validate a new phylogenomics strategy to efficiently select a minimal set of stable markers able to reconstruct the underlying species phylogeny. In contrast to previous approaches, our methodology does not only rely on the ability of individual genes to reconstruct a known phylogeny, but it also explores the combined power of sets of concatenated genes to accurately infer phylogenetic relationships of species not previously analyzed. We applied our approach to two broad sets of cyanobacterial and ascomycetous fungal species, and provide two minimal sets of six and four genes, respectively, necessary to fully resolve the target phylogenies. This approach paves the way for the informed selection of phylogenetic markers in the effort of reconstructing the tree of life.

## INTRODUCTION

Evolutionary relationships among species have been traditionally inferred using ribosomal genes, especially the 16S ribosomal DNA, due to their ubiquity, ease of amplification and appropriate level of conservation for most purposes. Many other genetic markers have been introduced, some of them specific for certain taxonomic groups (e.g. *rbcL* in plants) or only suitable for a certain taxonomic level, e.g. intraspecific analyses versus phylogenies at the level of orders or above (1). However, the actual usefulness of a specific gene for phylogenetic purposes could often be verified only by trial and error—after amplification, sequencing and phylogenetic analysis. To make a comparison of newly generated sequence data with data from previous studies possible, phylogenetic usefulness might have been sacrificed. However, with the increasing availability of completely sequenced genomes, we have now a whole range of genes at our disposal. Several phylogenomics approaches aim at using most of the information available on sets of complete genome sequences to derive a species phylogeny (2), or to investigate the variability among individual gene trees (3–6). Nonetheless, there is still the need to select phylogenetic marker genes to target unsequenced species. This asks an important question: which combination of genes is the most informative to establish the phylogenetic relationships of a given group of organisms. In this context, earlier work has focused on ranking phylogenetically informative genes based on their ability of reconstructing a known species phylogeny (7,8). The assumption is that genes that carry sufficient information to reconstruct the known part of the phylogeny are expected to perform similarly well in so far unsampled regions of the tree. However, this assumption is usually not proven within the framework of phylogenetic marker selection. An additional limitation of current marker selection procedures is that individual genes, rather than combinations of genes, are ranked. Previous studies have shown that different genes may be better suited to resolve different parts of the phylogeny (9,10), and hence it is important to consider the resolving power of combinations of marker genes. Ideally, an informative set of genes should be present in the studied species and remain informative when more taxa are added to the study. In addition, to limit costs of targeted sequencing, this set should be of a minimal possible size, but of sufficient size to carry enough information to reconstruct a phylogeny that goes beyond the one used during the selection phase.

To address these limitations, we present here a method to automatically identify, from whole-genome sequences small subsets of widespread genes that can accurately reconstruct the target phylogeny. In contrast to previous methods, our approach ranks combinations of genes, rather than individual loci. In addition, our approach includes a validation phase to ensure high accuracy when using species not considered for the marker selection, thus better reflecting real scenarios. To validate our method, we applied it to the selection of phylogenetic marker genes in a prokaryotic group—Cyanobacteria—and a eukaryotic group—Ascomycota (Fungi). Our results indicate that small sets of six and four genes, respectively, are able to precisely recover the target phylogenies, even after including additional species not used for the selection of markers.

## MATERIALS AND METHODS

### Sequence data

Proteins encoded in 63 and 83 completely sequenced genomes from Cyanobacteria and Ascomycota, respectively, were downloaded from various sources (Supplementary Tables S1 and S2). Additionally, 28 completely sequenced genomes (Supplementary Table S3) from Basidiomycota (Fungi) were downloaded from various sources for an additional validation test.

### Construction of training and testing sets for marker selection and validation

Available genomes were split into two sets: (i) the training set (T-set), accounting for around two-third of the available genomes, which was used to identify potential marker genes, and (ii) the validation set (V-set), comprising the remaining one-third of the genomes, which was used to evaluate whether marker genes were widespread and phylogenetically informative when species not included in the selection of markers are included. The composition of both sets was determined randomly, but a subsequent manual inspection phase ensured that representatives for all the main taxonomic groups were present in both the training and the validation sets. An alternative partitioning of the data with each set including half of the genomes was also tested.

### Selection and alignment of widespread single-copy gene families

A first step in the selection process identifies widespread genes present in single copy in all genomes in the T-set. This is done by performing a BLAST (11) search from a seed species against all other genomes, and selecting those proteins with a single hit (e-value cut-off 10^−^^5^ and coverage >50%) in every other genome. The cut-off in terms of number of species in which the marker should be present could be relaxed if a limited number of genes fulfill this criterion. The selection of the seed species is arbitrary, and more than one seed can be used to increase the number of detectable single-copy proteins. Here, we selected multiple seed species, one from each of the four and five major phylogenetic groups in Cyanobacteria and Ascomycota, respectively (species used as a seed are indicated in Supplementary Tables S1 and S2, respectively, with grey boxes). Each seed species defines a set of widespread proteins, which may overlap significantly with those obtained from the other species. If the union or, alternatively, the intersection of all sequences provides a number of markers considered sufficiently large, then it, is used as the initial set. These orthologous groups were aligned using the pipeline described in (12). In brief, sequences were aligned using three different programs: MUSCLE v3.8 (13), MAFFT v6.712b (14) and DiAlign-TX (15). Alignments were performed in forward and reverse orientation [i.e. using the Head or Tail approach (16)], and the six resulting alignments were combined into a consensus alignment using M-Coffee (17). The resulting combined alignment was subsequently trimmed with trimAl v1.4 (18), with a consistency score cut-off of 0.1667 and a gap score cut-off of 0.1, to remove poorly aligned regions.

### Phylogenetic tree reconstruction

Phylogenetic trees based on a Maximum Likelihood (ML) approach were inferred from individual or concatenated alignments. ML trees were reconstructed using the best-fitting evolutionary model, which was selected as follows: A phylogenetic tree was reconstructed using a Neighbour Joining (NJ) approach as implemented in BioNJ (19). The likelihood of this topology was computed, allowing branch-length optimization, using seven different models (JTT, LG, WAG, Blosum62, MtREV, VT and Dayhoff), as implemented in PhyML v3.0 (20). The two evolutionary models best fitting the data were determined by comparing the likelihood of the used models according to the AIC criterion (21). Then, ML trees were derived using these two models and the one with the best likelihood was used for further analyses. The topological rearrangement method was set to either Nearest Neighbor Interchange—to infer the phylogenies for individual alignments—or Subtree Pruning and Regrafting in the case of analyses of concatenated alignments. In all cases, a discrete gamma-distribution with four rate categories plus invariant positions was used, estimating the gamma parameter and the fraction of invariant positions from the data.

### Assessment of informativeness for phylogenetic reconstruction

The ability to reconstruct the reference phylogeny was used to rank individual reconstructions by comparing them to a reference species phylogeny. For this, a reference species phylogeny is reconstructed from the concatenation of all individual alignments. Then, each individual or concatenated alignment is scored according to its ability to reconstruct the reference phylogeny using the distance measure of choice. By default, we used the Robinson and Foulds (RF) distance (22) as implemented in Ktreedist (23), but other distance measures could be used, for instance, the Tree Certainty (10), the Nodal Distance (24) or the Likelihood ratio, as described in (25) between each individual tree and the reference one. We here tested the performance of several of them.

### Selection of combined minimal sets of phylogenetic marker genes

**Figure 1.**
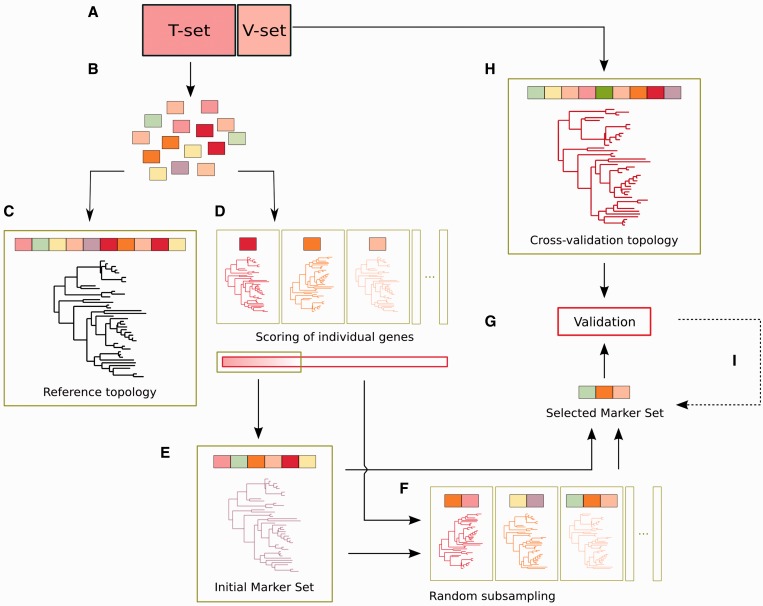
Schematic representation of the proposed marker selection pipeline, including the training and the validation steps. (**A**) The complete set of available genomes is divided into a training and validation set. (**B**) For the validation set, orthologous groups of genes present in single copy in all the species are selected and aligned. (**C**) A phylogeny reconstructed from the analysis of the concatenated alignments is considered as the reference topology. (**D**) Individual alignments are also used to build individual gene phylogenies. The similarity of these phylogenies to the reference topology is used to score the phylogenetic informativeness of the genes, which are ranked accordingly. (**E**) Top-scoring genes are concatenated sequentially until the resulting alignment yields a phylogeny identical (or sufficiently similar according to a given similarity threshold) to the reference topology. The gene families in such a concatenated alignment constitute the ‘initial marker set’, which is sufficient to obtain the desired level of resolution; as indicated, it is possible to move directly to the validation phase or to optimize the markers set size. (**F**) Smaller sets of marker genes with resolving power equal to the initial marker set are searched for iteratively by random subsampling of available markers either from the whole set or from the current set of markers, and evaluation of the resulting phylogenies. The process is finished when a sufficiently small marker set with sufficiently high resolution power is found or, alternatively, when the full combinatorial space has been explored. (**G**) Selected marker gene sets are validated against reference topologies that include species of the validation set. (**H** and **I**) Alternative sets of marker genes can be tested if previously selected ones fail during the validation phase.

A two-step procedure is used to find combined sets of marker genes of a minimal size (see Figure 1). Firstly, alignments of the widespread genes are concatenated progressively in decreasing order of their phylogenetic informativeness score (i.e. increasing order of their RF distance against the reference topology). That is, the *n* top-scoring markers are concatenated and used to reconstruct a species phylogeny (see below). This is, repeated from *n* = 2 to *n* = *m*, *m* being the minimal number of concatenated marker genes that reach a cut-off distance; to the reference tree (we here used a RF distance cut-off of 0). This set of *m* marker genes is referred to as the ‘initial marker set’. Secondly, if the ‘initial marker set’ is considered too big, another iterative phase is started to find subsets of size smaller than *m*, which nevertheless reach the same cut-off distance. To do so, sets of size, ranging from 2 to *m −* *1*, are formed by randomly subsampling genes from the whole set of marker genes. Each subset is evaluated in terms of its phylogenetic informativeness. This iterative process finishes when (i) all possible combinations have been explored, (ii) at least one smaller combination with the same cut-off has been found or (iii) a number of predefined iterations has been reached (here we explored a minimum of 100 combinations). When one smaller combination is found, the iterative process can be restarted, setting that combination as the ‘initial marker set’. In the case of exploring all possible combinations without finding a smaller set of genes, the ‘initial marker set’ is returned as the minimum possible concatenation of individual gene sets that recovers the reference tree. If more than one iteration is performed to reduce the ‘initial marker set’ size, it is possible to constrain the random subsampling to the initial marker set to refine the current selection rather than considering alternative markers from the whole data set.

### Validation

The ability of the selected marker sets to properly reconstruct the phylogeny when including species not present in the training phase is validated. For this, homologs of the widespread genes are identified in all genomes of the V-set. Then two ‘hold out’ validation tests are applied.

A) One-species-at-the-time test: For each genome in the V-test, the reference tree is expanded by adding the corresponding homologs of the widespread genes, and following the procedure described above. This expanded reference is used to evaluate the ability of the marker gene set to correctly position the new species.

B) Cross-validation: In this second test, only the new set of genomes (the V-set) is used to derive a new reference topology and the tree based on the set of marker genes. Then, both topologies are compared to evaluate the ability of the set of marker genes to recover the new reference topology.

### Code availability

Necessary scripts to implement the pipeline can be accessed here: http://github.com/scapella/markers_genes.

## RESULTS AND DISCUSSION

### From individual gene markers to combined sets

The rationale behind the proposed methodology is that phylogenetic gene markers are generally used in combination, rather than individually, and that their performance to reconstruct accurate phylogenies should be evaluated beyond the set of species used for their prioritization. Like other recently developed genome-wide methods (7), our procedure starts by evaluating the ability of single gene trees to recover a reference species phylogeny. This produces a ranked list of marker genes. While other procedures stop there, ours goes one step further and evaluates combinatorial sets of marker genes. This is done using a multi-attribute optimization of two conflicting criteria: a minimal gene size, and maximal information content. A final cross validation step evaluates the performance of such selected gene marker sets to reconstruct accurate phylogenies including species not previously seen in the selection phase.

In brief, our proposed pipeline proceeds as follows (see Figure 1, pseudocode is provided in the Supplementary Material Section S3, and additional details are provided in the ‘Materials and Methods’ section): (i) Available genomes are divided into two non-overlapping sets: the training set (T-set), which will be used for marker selection, and the validation set (V-set) used in a later stage; (ii) Widespread genes with single-copy orthologs in all genomes of the T-set are selected using a blast-based strategy, each orthologous group is aligned; (iii) the concatenation of all such alignments is used to reconstruct a phylogeny that will be considered as the reference; (iv) each individual alignment is also used to reconstruct a single gene tree, which is compared with the reference tree. The smaller the difference between the two topologies, the higher is the informativeness score of that orthologous group; (v) alignments of orthologous groups are concatenated sequentially, in a decreasing order of their scores, and used to reconstruct a phylogeny, until a desired similarity to the reference tree is achieved. The concatenated genes at this point constitute the initial marker set; (vi) subsets smaller than the initial marker set are selected randomly from the whole set of marker genes and their ability to recover the reference phylogeny evaluated. This process is repeated iteratively until all combinations are explored or a desired size and informativeness of the set of gene markers is achieved. As a result a set of marker genes is selected, (vii) in a final step the set of selected marker genes is evaluated for its ability to reconstruct an accurate topology when the genomes of the validation step are included. This constitutes the backbone of the proposed strategy, which can be implemented with different particular methodologies for the selection of orthologs, alignments of sequences, reconstruction of phylogenies, measurement of topological differences, etc. We applied this procedure to available genome sets of Cyanobacteria and Acomycota fungi (see below). This pipeline is amenable to parallelization: the computational cost of running the pipeline in the Ascomycota data set was ∼8 h in a 500-nodes computing cluster, which is equivalent to 175 days in a single processor.

### Six gene markers for Cyanobacteria phylogeny

Cyanobacteria are prokaryotes capable of oxygenic photosynthesis, and the origin of the chloroplasts of today’s green plants. Being ∼3.5 billion years old (26), they now inhabit all ecosystems and continents on earth, including the Antarctic. Taxonomy and phylogeny were always challenging in the Cyanobacteria. For prokaryotes, they are comparatively feature-rich in their morphology, but still the number of morphological traits is insufficient to provide enough information for a phylogenetic analysis. Already the first molecular analyses based on 16S rDNA only suggested that the traditional classification of Cyanobacteria is highly artificial (27). Although the 16S rDNA is still the most common phylogenetic marker in Cyanobacteria, other genes have been used to generate phylogenies at various taxonomic levels, e.g. gyrB, rpoC1, rpoD1 (28), nifD (29) and others [see (30) for an overview]. However, the availability of specific single locus data varies tremendously across taxa and species, and the number of taxa for which sequence data is available decreases quickly as the number of loci increases. As a result, data sets with larger numbers of loci often include only few cyanobacterial taxa, and vice versa. Large-scale phylogenies using multiple gene loci as data source are restricted to taxa for which full genome data are available (31,32).

**Figure 2.**
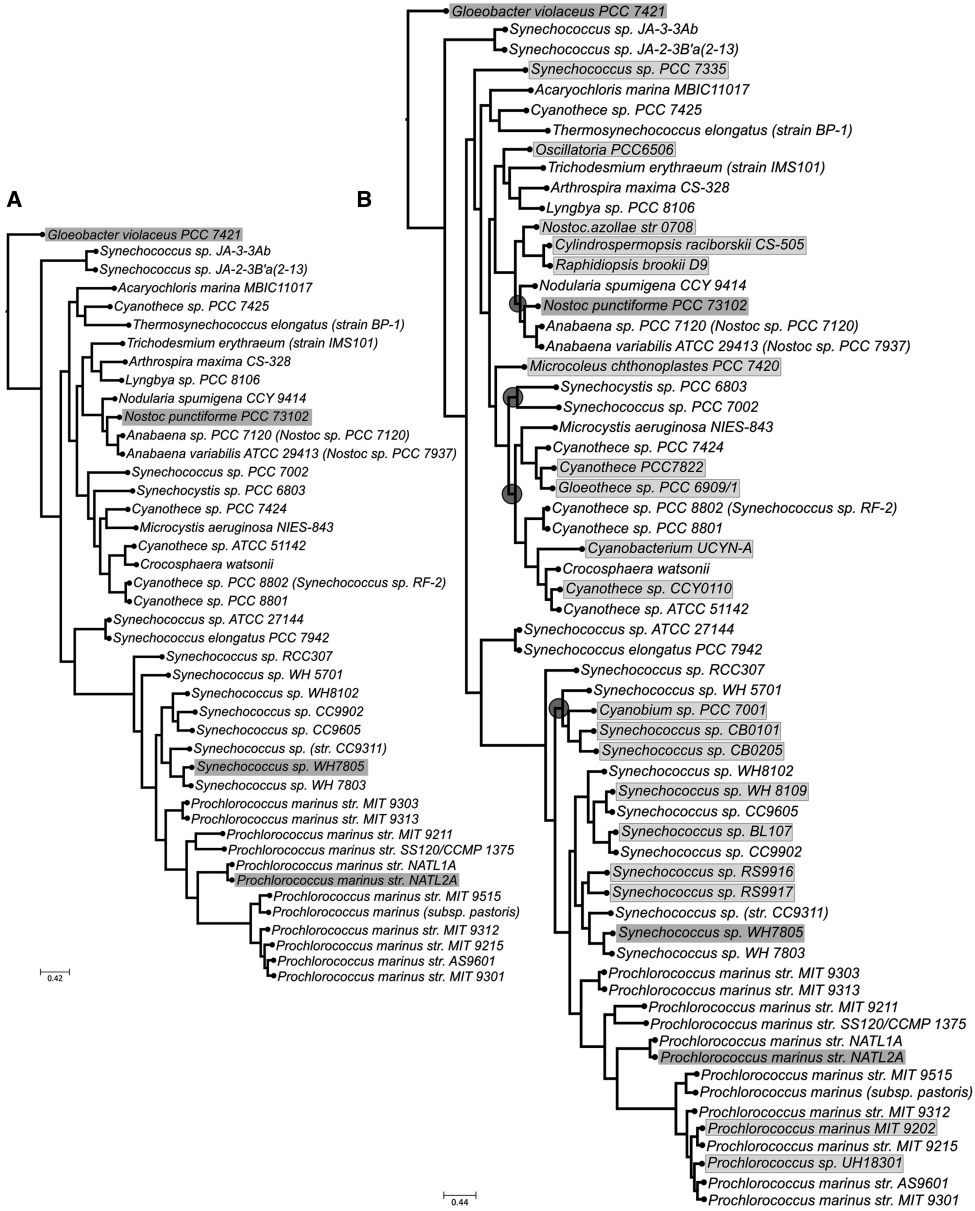
Cyanobacterial phylogenies comprising different sets of species. (**A**) Reference tree for the training phase, comprising 43 species. Grey boxes indicate which species were used as seed to perform the BLAST searches. (**B**) Tree inferred using the concatenation of all available gene markers for the 62 species used in this study. Grey boxes indicate which species were used as query to perform the BLAST searches during the training phase. Light grey boxes indicate species which belong to the validation set. Dark grey circles indicate conflicts between this tree and the one reconstructed using only the proposed six gene markers. Chi-square–based parametric branch supports were computed using an approximate likelihood ratio test and are not shown in the tree because all of them are equal to 1.

**Figure 3.**
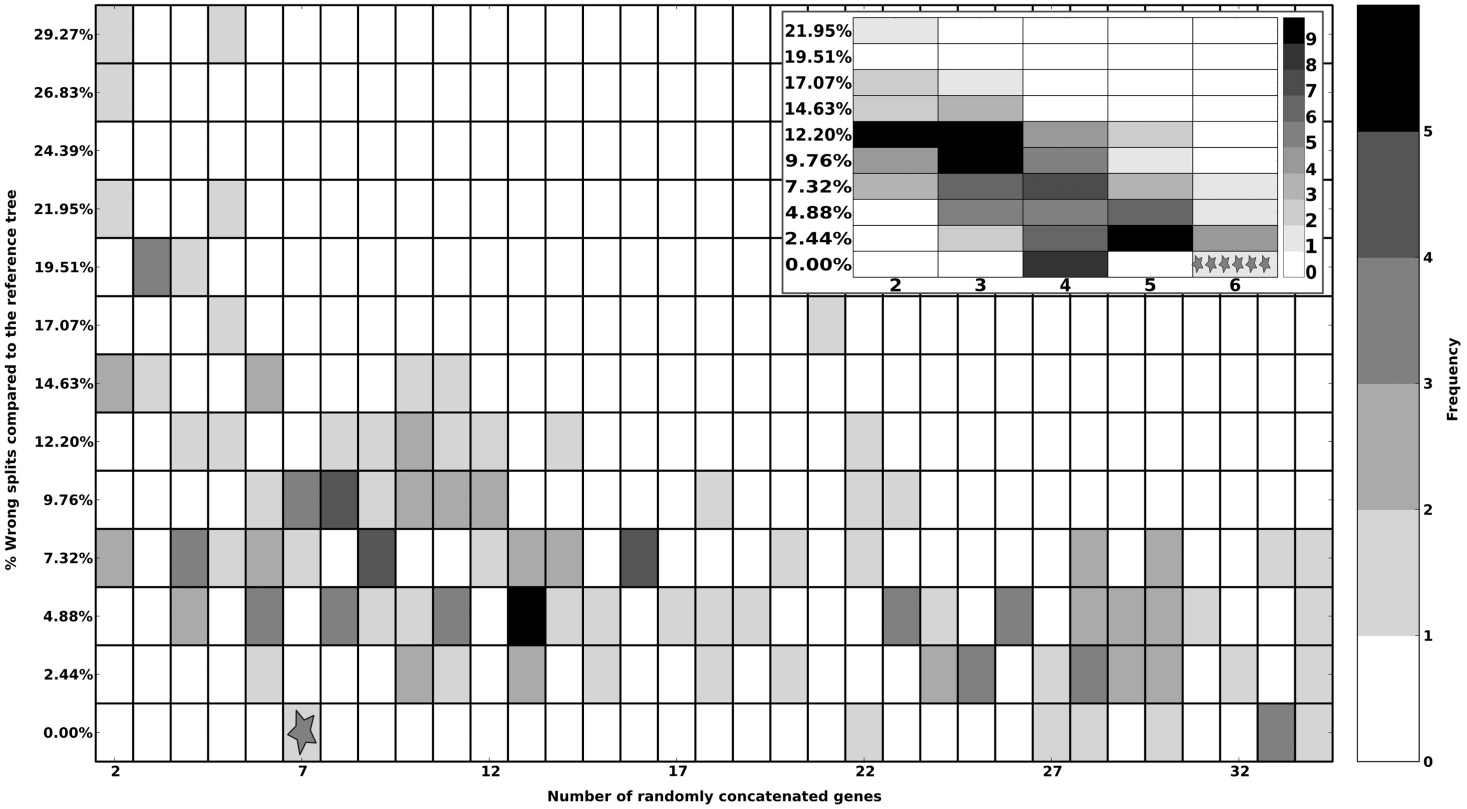
Performance of the different randomly selected subsets of up to 34 proteins in terms of percentage of wrong splits (normalized RF distance) when compared with the reference species tree. One hundred forty-seven sets were generated to find a combination smaller than the initial set of marker genes (35 genes). The smallest combination of genes that recover the reference ascomycetes fungal tree of life is marked in the plot with a star. Inset figure shows the performance of all possible subsets of size two to six of the selected set of seven marker genes. Although many subsets of size four recover the reference topology on this phase, a set of six marker genes was selected giving their performance across all tests (marked with stars).

We applied our proposed method to identify reliable sets of markers genes using 62 species with completely sequenced genomes (Supplementary Table S1). In the training phase, a reference tree for 43 species was inferred using a concatenated alignment of 203 single-copy genes present in all species (Figure 2, panel A). For the shared species, this tree is fully congruent with a recent phylogeny based on 340 genes (31), except for the relative positions of *Acaryochloris marina* and *Thermosynechococcus elongatus*. Our pipeline defined an initial marker set of 35 genes able to fully recover the reference phylogeny (see Supplementary Figure S1). The iterative search for smaller sets with an equal phylogenetic potential yielded a subset of seven genes (see Figure 3). An additional iteration was performed to reduce this marker gene set. For this, all possible combinations of the seven genes in subsets of size two to six were explored (see inset Figure 3) leading to a smaller set of six genes (see Table 1). Validation on the remaining 19 genomes (V-set, Figure 2B light grey boxed) showed that, in all cases, the marker genes found (6 in 17 genomes, 5 in 2 genomes) were able to accurately place the test species when used in combination. Of note, in eight cases the resulting topology included some minor differences affecting other parts of the tree (up to 4.7% different splits) (see Supplementary Table S4). Finally, when the six marker genes were used to reconstruct the 63-species phylogeny including all genomes in the training and test sets, they resulted in a topology largely similar to the reference tree (Figure 2B) except for four conflicting nodes, of which one is due to a change in the arrangement of strains of the same species. Finally, the topological accuracy measured with the tree certainty score (10) was higher for the tree inferred from the selected marker genes (18.06) than for any tree inferred from individual genes (average 8.17, maximal value 17.28).

**Table 1.** List of selected phylogenetic marker genes in Cyanobacteria

Uniprot Id	Length (AA)	Annotation
B2IVU1	246	Probable 2-phosphosulfolactate phosphatase.
B2J427	979	Glycine dehydrogenase [decarboxylating]
B2IT89	480	Trigger factor
B2IW68	816	Phenylalanyl-tRNA synthetase, beta subunit
B2J6R0	312	Cytochrome oxidase assembly
B2J980	1087	Carbamoyl-phosphate synthase, large subunit

Protein information has been taken for *Nostoc punctiforme*.

To compare the performance in the cross-validation of our set of marker genes against commonly used protein-coding markers, we selected those markers proposed in (28) and (29). Using the same homology search strategy, we scanned the complete proteomes of the 62 species for gyrB, rpoC1, rpoD1 and nifD genes. NifD was found to be single copy in only 23 out of the 62 scanned proteomes owing to the presence of duplications, low coverage between query and target proteins or absence of any significant hit. Thus, two data sets were prepared, one with the concatenation of the above-mentioned four genes, and another one excluding nifD, for the three possible data sets (training set, validation set and all species). Then, reconstructed trees were compared with species reference trees using all available markers (see Supplementary Table S5). In this case, our set of six marker genes performed better than the concatenation of the commonly used genes because the latter never recovered the reference species tree topologies (up to 26.7% of wrong splits when considering all species). To discard the possibility that this better performance was simply due to the larger size of our set (six genes versus four traditional markers), we evaluated all possible subsets of three and four genes from our set of markers. Most of them (68 over 70) performed better than any of the two combinations of traditional markers (see Supplementary Table S6), indicating a higher performance of the markers selected by our procedure. However, none of these smaller subsets displayed an overall higher performance than the six-genes set, and therefore were not selected as the final set of marker genes.

### Four gene markers for the fungal tree of life

**Figure 4.**
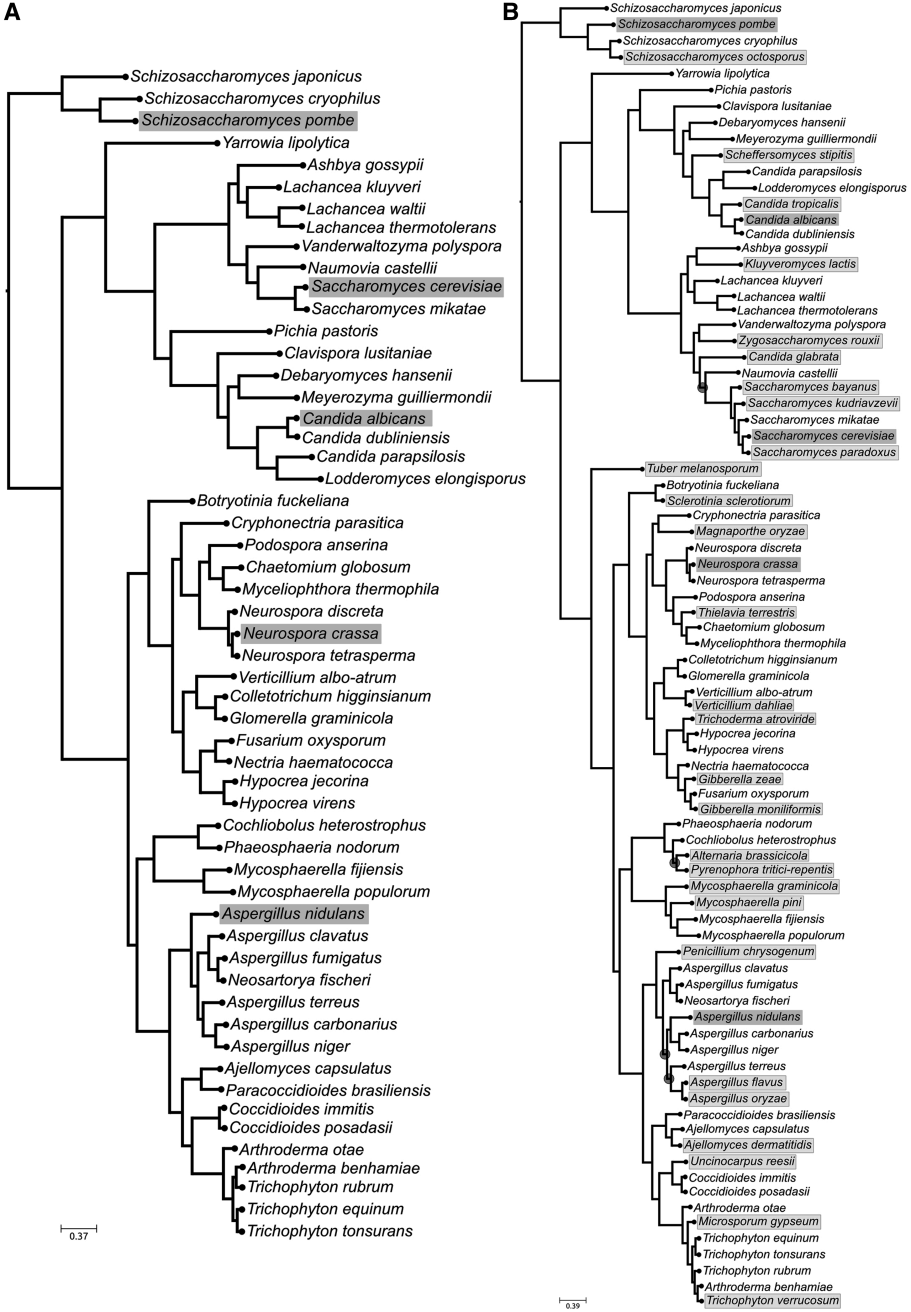
Ascomycota fungal phylogenetic trees comprising different sets of species. (**A**) Reference tree for the training phase, comprising 55 species. Grey boxes indicate which species were used as seed to perform the BLAST searches. (**B**) Tree inferred using the concatenation of all available gene markers for the 83 species used for the analyses. Grey boxes indicate which species were used as query to perform the BLAST searches during the training phase. Light grey boxes indicate species which belong to the validation set. Dark grey circles indicate conflicts between this tree and the one reconstructed using only the proposed four gene markers. Chi-square–based parametric branch supports were computed using an approximate likelihood ratio test and are not shown in the tree because all of them are equal to 1.

With estimated 1.5 million species (33), fungi constitutes one of the most diverse eukaryotic groups. In addition, their generally unicellular organization and their broad phenotypic and metabolic plasticity makes genetic approaches the best suited for establishing fungal diversity and phylogenetic relationships. Previous studies to establish phylogenetic relationships in fungi have used widespread gene markers such as subunits 1 and 2 of RNA polymerase II (RPB1, RPB2), elongation factor 1α (ef1α) and β-tubulin (β-tub) (34,35). In addition, as a result of the growing availability of fully sequenced fungi, genome-wide approaches are increasingly being used (36–38). Despite large international initiatives to sequence thousands of fungal genomes (e.g. http://1000.fungalgenomes.org), the need for phylogenetic markers to target a broader diversity as well as unculturable species will still exist for the coming years. We thus applied our approach to select stable phylogenetic markers using 83 available fungal genomes belonging to the ascomycetes (Supplementary Table S2). A reference phylogeny based on 169 widespread single-copy genes of the 55 species in the training set is largely congruent, for the shared species, with earlier reconstructed trees (36–39) (see Figure 4 panel A). The sequential concatenation of markers in decreasing order of their phylogenetic informativeness defines an initial marker set of six genes to accurately recover the reference topology when using RF (22) as distance measurement (see Supplementary Figure S2). The subsequent sampling and testing of subsets reduces the number of necessary markers to only four genes (see Supplementary Figure S3 and Table 2). This number is smaller than the six-gene marker set used in previous large-scale phylogenetic surveys of fungi (35,40). A validation of this set of marker genes in the V-set, comprising 28 species, showed that in most cases (25 genomes) the four genes could be found in single copy, while in three genomes one of the marker genes was missing or present in multiple copies. In all cases the marker gene set was able to reconstruct an expanded phylogeny with <6% of wrong splits, being fully congruent in 14 (50%) of the species (see Supplementary Table S7). Similar to the cyanobacterial case, the topological accuracy measured with the tree certainty score (10) was higher for the tree inferred from the selected marker genes (34.34) than for any tree inferred from individual genes (average 28.61, maximal value 31.30).

**Table 2.** List of selected phylogenetic marker genes in Ascomycota

Uniprot Id	Length (AA)	Annotation
YHR186C	1557	Target of rapamycin complex 1 subunit KOG1
YMR012W	1277	Clustered mitochondria protein 1
YJL029C	822	Vacuolar protein sorting-associated protein 53
YAR007C	621	Replication factor A protein 1

Protein information is related to *Saccharomyces cerevisiae*.

In the second test performed using only the genomes from the validation set, full agreement was found between the trees derived using either the complete sets of single-copy genes or just the set of marker genes. To validate our marker genes selection, we took four protein-coding genes often used as markers to resolve the fungal tree of life: tef1 (elongation factor 1α), tub2 (β-tubulin), tsr1 and cdc47p. The latter two correspond to the marker genes proposed by (7). We prepared two data sets, one containing the concatenation of the four above-mentioned proteins, and the second one containing only the two genes, which have been proposed to resolve the fungal species tree (see Supplementary Table S8). None of these data sets were able to recover the same tree topology as compared with the tree derived using all available markers for the training, the validation or the complete sets of species with a percentage of wrong splits up to 14.81%. Finally, we compared the performance of our selected set of four genes in the V-set, with a random sampling of 1000 different sets of size four. Only 2 of the 1000 randomly chosen sets (0.2%) reached the same distance (RF = 0) to the reference tree as our selected set (see Supplementary Figure S4).

We measured the sensitivity of our pipeline to the initial partitioning of species among T and V sets. For this we performed three additional runs to search for Ascomycotina marker sets. On two of these additional runs (Ascomycota 2 and 3) different species sets were used in the described 2:1 ratio, while for the third run (Ascomycota 4) the ratio of species between the T and V sets was set to 1:1 (see Supplementary Table S2). We found a high correlation between the scores of individual genes derived in the runs with the same number of species (Ascomycota 2 and 3) (>0.7, Pearson correlation coefficient). However, each of these runs converged in different selected sets of marker genes (Supplementary Table S9). Note that this result is not unexpected because our procedure includes random sampling and does not involve an exhaustive search among all possible combinations. Even different repetitions over the same initial species partitioning may result in different selected markers. Thus simple repetitions of the procedure or iterations over possibly different initial partitions may serve to find alternative marker gene sets with a similar resolutive power. Altering the partitioning scheme so that T-set and V-set are equally large (1:1, Ascomycota 4) had the expected results: Because less species are used in the T-sets, many more widespread genes are found, then, typically smaller sets are able to recover the reference topology. However, given that the V-set is larger, many of the selected sets do not pass the validation phase, either because they are not widespread among the species in the V-set or because they do not reach sufficient similarity to the reference topology in the cross-validation. Thus, different partitioning schemes can influence the search for suitable markers, and we have found that the 2:1 scheme, typically used in many machine-learning approaches, performed well in the two data sets used. Finally, we explored four alternative measures of topological distances, the so-called nodal distance (24) to the reference tree, the recently proposed tree certainty score considering the whole set of single-gene trees (10), the K-tree score (23), which considers also the branch lengths among trees being compared, and the use of the likelihood ratio (25) between the two compared trees. Both topology-only measures (nodal distance and certainty-score) were highly correlated with the RF distance (Supplementary Table S10). In contrast, K-tree score and likelihood ratios were poorly correlated with RF distances, as expected given the relevance of branch lengths in addition to topology in these scores (Supplementary Table S10). Given this discordancy in the ranking of marker genes we further explore the process of marker selection using these two alternative measures as our ranking scheme. K-tree scores needed the concatenation of 42 genes before the reference topology was recovered, and thus we conclude it is not a good measure to select gene marker sets aiming to recover a target topology. The use of likelihood ratios initially provided promising results with the concatenation of the four top-scoring genes readily recovering the target topology. However, this set provided poor results in the cross-validation phase, suggesting that this approach may possibly be overfitted to the training set, and does not result in marker sets able to recover phylogenies outside the training set. Thus, among the distance measures explored RF, certainty score and nodal distance seem to provide congruent and satisfactory results.

Finally, to measure the performance of the four selected gene markers for reconstructing accurate phylogenies outside the particular group used to select the markers (ascomycetes), we tested their performance to reconstruct a phylogeny for basidiomycetes. For this we used 28 fully sequenced Basidiomycota species (see Supplementary Table S3), and used the approach described above to reconstruct a reference phylogeny based on 313 genes. Despite having been selected from within ascomycete genomes, the four marker genes produced a phylogeny for basidiomycetes that was fully congruent with the reference (see Supplementary Figure S5). Altogether our results show that the four selected gene markers, used in combination, have a strong potential to reconstruct accurate phylogenies of fungal species and that they will be valuable in the expansion of the fungal tree of life. In contrast, the use of a three-gene marker set selected for basidiomycetes did not resolve the ascomycetes phylogeny (results not shown). We interpret this difference in the light of the clear unbalance of taxonomic sampling across the two fungal lineages. Ascomycetes are well sampled and a high number of genomes are available (83 genomes). As a result the initial set of marker genes is relatively small (169), and only few combinations are able to resolve the target phylogeny. In contrast, basidiomycetes are sparsely sampled (28 genomes), the initial set of gene markers is big (552) and many potential combinations of few genes resolve fully the simple target phylogeny. Thus, it seems likely that the number and diversity of the available genomes affects the potential of the selected gene markers to accurately work outside the tested lineages.

## CONCLUSION

Reconstructing the tree of life is a daunting task that will require the combination of diverse efforts and methodologies. It is most likely that the expansion of the tree of life and the increase in resolution will proceed through the agglutination of several studies. Some, based on complete genomes, will establish a backbone of the main lineages, while others, more focused studies, will resolve internal diversity within a specific clade based on targeted markers. In addition, the expansion of the tree of life towards less-explored clades will likely proceed in a two-step manner. First, an overview of phylogenetic relationships within the new clades will be sketched through targeted amplification and analysis of selected phylogenetic markers. Second, based on these results, several species will be selected for complete sequencing to provide a first backbone of the new clade, from which to continue with targeted sequencing analyses. In all these contexts, the informed selection of phylogenetic marker genes constitutes a necessary step. Previous efforts have focused on finding widespread genes in relatively large clades (41), or combining those with the potential for reconstructing a reference phylogeny (7). Recent analyses have shown that careful selection of genes based on their gene phylogeny provides more accurate results than simply concatenating a large number of genes (10). This is a logical outcome of the fact that different genes evolve at different rates and are thus expected to provide different resolution at different tree depths (9). Similarly, evolutionary rates, but also rates of gene duplication, loss or horizontal transfer may vary across the represented lineages. Thus, an empirical approach to select good phylogenetic markers should ideally consider sets of phylogenetic gene markers, rather than individual genes, and should check that their resolutive power remains high when additional, previously unseen taxa are considered. Here, we have developed a new approach that is based on the selection of sets of marker genes from completely sequenced genomes based on their combined power to resolve a reference phylogeny. The assessment of a combination of genes, rather than individually, constitutes one of the major novelties of the proposed approach. This, in our view better reflects current scenarios in which several, rather than a single, phylogenetic markers are obtained from a set of selected species. In addition, as we have shown here for Cyanobacteria and Fungi, the exploration of combinatorial effects of the concatenation of good phylogenetic markers is able to reduce the number of selected markers while keeping a similar potential for phylogenetic reconstruction. Furthermore, our procedure comprises validation tests to assess the performance of the selected markers outside the genomes used in the selection of marker genes. This is, to the best of our knowledge, the first time that such a validation is built-in in the marker selection pipeline. As shown here, the validation test provides information on how the marker genes will behave when used on additional species, as well as an indication of how the resolving power may decrease when expanding the tree to include other species within the clade. These are important considerations for the selection of phylogenetic marker genes, and for which tools were lacking so far. Thus, our proposed approach fills in an important gap and constitutes a valuable tool in the informed selection of phylogenetic markers. We wish to note that our approach provides a general framework that is easily adapted to specific needs. For instance, when the aim is to maximize the resolution of specific internodes in the reference phylogeny, a collapsed or a weighted reference topology can be used to direct our selection of markers. Similarly alternative measures of phylogenetic informativeness, other than the distance to the reference phylogeny, could be used. Additional criteria, such as the suitability of markers for primer design and experimental amplification (8), are not specifically tackled here, but could be considered in a downstream analysis. Finally, although we have developed our approach with targeted gene sequencing approaches in mind, it can potentially be useful in other scenarios that may become relevant in the future, such as the selection of sequences from raw (meta)genome or transcriptome sequencing data, or the selection of a subset of representative genes for computationally costly analyses (e.g Bayesian inference).

## SUPPLEMENTARY DATA

Supplementary Data are available at NAR Online.

## FUNDING

Spanish ministry of science and innovation [BIO2012-37161 towards T.G. group research (in part)] and Qatar National Research Fund [NPRP 5-298-3-086]. Funding for open access charge: Core funding from the group of the corresponding author.

*Conflict of interest statement*. None declared.

## Supplementary Material

Supplementary Data
