# Supplementary material for: A phylogenomics approach for selecting robust sets of phylogenetic markers
Source: Nucleic Acids Res. 2014 Jan 28;42(7):e54. doi: 10.1093/nar/gku071 (PMC3985644; doi:10.1093/nar/gku071)
Supplement: Supplementary Data [file supp_42_7_e54__index.html]

A phylogenomics approach for selecting robust sets of phylogenetic markers — A phylogenomics approach for selecting robust sets of phylogenetic markers — Supplementary Data 

# A phylogenomics approach for selecting robust sets of phylogenetic markers

## Supplementary Data

files

**Files in this Data Supplement:**

- Supplementary Data - pdf file
