# Supplementary material for: A phylogenomics approach for selecting robust sets of phylogenetic markers
Source: Nucleic Acids Res. 2014 Jan 28;42(7):e54. doi: 10.1093/nar/gku071 (PMC3985644; doi:10.1093/nar/gku071)
Supplement: Supplementary Data [file supp_gku071_nar-01751-met-k-2013-File006.pdf]

Supplementary material for the article:

"A phylogenomics approach for selecting robust sets of phylogenetic markers"

Salvador Capella-Gutierrez, Frank Kauff, and Toni Gabaldón*

Content:

1. Supplementary tables.
2. Supplementary figures.
3. Pipeline description in pseudo-code.

1. Supplementary tables.

Table 1.

List of 63 completely sequenced Cyanobacterial genomes used in the analyses. Columns indicate, in this order, the taxonomic group, the species, the source from where proteomes were obtained, and date of acquisition. Gray boxes indicate which species were used as query in the orthology search. Yellow boxes indicate which species were used for the validation phase.

Taxonomic Group	Scientific Name	Source	As of
Chroococcales	*Microcystis aeruginosa (strain NIES-843)*	integr8	2010/01
Synechococcales	*Synechocystis sp. (strain PCC6803)*	integr8	2010/01
Chroococcales	*Crocosphaera watsonii (strain WH8501)*	integr8	2010/05
Chroococcales	*Cyanothece sp. (strain ATCC 51142)*	integr8	2010/01
Chroococcales	*Cyanothece sp. (strain PCC 7424)*	integr8	2010/01
Chroococcales	*Cyanothece sp. (str. PCC7425/ATCC29141)*	integr8	2010/05
Chroococcales	*Cyanothece sp. (strain PCC 8801)*	integr8	2010/05
Chroococcales	*Cyanothece sp. (strain PCC 8802)*	integr8	2010/01
Synechococcales	*Synechococcus elongatus*	integr8	2010/01
Synechococcales	*Synechococcus elongatus (str. PCC7942)*	integr8	2010/01
Synechococcales	*Synechococcus sp. (strain PCC6301)*	integr8	2010/05
Synechococcales	*Synechococcus sp. (strain PCC7002)*	integr8	2010/05
Synechococcales	*Synechococcus sp. (strain CC9311)*	integr8	2010/05
Synechococcales	*Synechococcus sp. (strain CC9605)*	integr8	2010/05
Synechococcales	*Synechococcus sp. (strain CC9902)*	integr8	2010/05
Synechococcales	*Synechococcus sp. (strain JA-2-3B)*	integr8	2010/01
Synechococcales	*Synechococcus sp. (strain JA-3-3Ab)*	integr8	2010/05
Synechococcales	*Synechococcus sp. (strain RCC307)*	integr8	2010/01
Synechococcales	*Synechococcus sp. (strain WH7803)*	integr8	2010/01
Synechococcales	*Synechococcus sp. (strain WH7805)*	integr8	2010/05
Synechococcales	*Synechococcus sp. (strain WH8102)*	integr8	2010/05

Synechococcales	*Synechococcus sp. (strain WH5701)*	integr8	2010/05
Chroococcales	*Cyanobacterium UCYN-A NCBI 2011/03*	NCBI	2011/03
Synechococcales	*Cyanobium sp. (strain PCC7001)*	NCBI	2011/03
Chroococcales	*Cyanothece sp. (strain CCY0110)*	NCBI	2011/03
Chroococcales	*Cyanothece (strain PCC7822)*	NCBI	2011/03
Chroococcales	*Gloeothece sp. (strain PCC6909/1)*	NCBI	2011/03
Synechococcales	*Synechococcus sp. (strain WH8109)*	NCBI	2011/03
Synechococcales	*Synechococcus sp. (strain PCC7335)*	NCBI	2011/03
Synechococcales	*Synechococcus sp. (strain RS9916)*	NCBI	2011/03
Synechococcales	*Synechococcus sp. (strain RS9917)*	NCBI	2011/03
Synechococcales	*Synechococcus sp. (strain CB0101)*	NCBI	2011/03
Synechococcales	*Synechococcus sp. (strain CB0205)*	NCBI	2011/03
Synechococcales	*Synechococcus sp. (strain BL107)*	NCBI	2011/03
Gloeobacterales	*Gloeobacter violaceus*	integr8	2010/01
Nostocales	*Nodularia spumigena (strain CCY9414)*	integr8	2010/05
Nostocales	*Nostoc punctiforme (strain PCC73102)*	integr8	2010/01
Nostocales	*Anabaena sp. (strain PCC7120)*	integr8	2010/01
Nostocales	*Anabaena variabilis (strain PCC7937)*	integr8	2010/01
Nostocales	*Raphidiopsis brookii (strain D9)*	NCBI	2011/03
Nostocales	*Nostoc azollae (strain 0708)*	NCBI	2011/03
Nostocales	*Cylindrospermopsis raciborskii (str.CS-505)*	NCBI	2011/03
Oscillatoriales	*Lyngbya sp. (strain PCC8106)*	integr8	2010/05
Oscillatoriales	*Arthrospira maxima (strain CS-328)*	integr8	2010/05
Oscillatoriales	*Trichodesmium erythraeum (str. IMS101)*	integr8	2010/01
Oscillatoriales	*Microcoleus chthonoplastes (str. PCC7420)*	NCBI	2011/03
Oscillatoriales	*Oscillatoria (strain PCC6506)*	NCBI	2011/03
Synechococcales	*Prochlorococcus marinus (strain AS9601)*	integr8	2010/01
Synechococcales	*Prochlorococcus marinus (str. MIT 9211)*	integr8	2010/05
Synechococcales	*Prochlorococcus marinus (str. MIT 9215)*	integr8	2010/05
Synechococcales	*Prochlorococcus marinus (str. MIT 9301)*	integr8	2010/01
Synechococcales	*Prochlorococcus marinus (str. MIT 9303)*	integr8	2010/01

Synechococcales	*Prochlorococcus marinus (str. MIT 9312)*	integr8	2010/05
Synechococcales	*Prochlorococcus marinus (str. MIT 9313)*	integr8	2010/05
Synechococcales	*Prochlorococcus marinus (str. MIT 9515)*	integr8	2010/05
Synechococcales	*Prochlorococcus marinus (str. NATL1A)*	integr8	2010/05
Synechococcales	*Prochlorococcus marinus (str. NATL2A)*	integr8	2010/05
Synechococcales	*Prochlorococcus marinus subsp. marinus str. CCMP1375*	integr8	2010/05
Synechococcales	*Prochlorococcus marinus subsp. pastoris (strain CCMP1986 / MED4)*	integr8	2010/05
Synechococcales	*Prochlorococcus marinus (str. MIT9202)*	NCBI	2011/03
Synechococcales	*Prochlorococcus sp. (str. UH18301)*	NCBI	2011/03

Table 2.

List of 83 completely sequenced Ascomycota genomes used in this study. Columns indicate, in this order, the taxonomic group, the species name, test rounds in which these species were used as part of the validation set or as queries for the orthology search, the source from where proteomes were obtained, and the date of acquisition. Gray boxes indicate which species were used as queries for the orthology search. Yellow boxes indicate which species were part of the validation set.

Taxonomic Group	Scientific Name	Round				Source	As of
		1	2	3	4		
Eurotiomycetes	*Arthroderma benhamiae*					The Broad Institute	2011/06
Dothideomycetes	*Alternaria brassicicola*					JGI	2011/06
Eurotiomycetes	*Aspergillus clavatus*					JGI	2011/06
Eurotiomycetes	*Aspergillus carbonarius*					JGI	2011/06
Pezizomycotina	*Ajellomyces capsulatus*					JGI	2011/06
Eurotiomycetes	*Aspergillus flavus*					JGI	2011/06
Eurotiomycetes	*Aspergillus fumigatus*					The Broad Institute	2011/06
Eurotiomycetes	*Aspergillus niger*					JGI	2011/06
Eurotiomycetes	*Aspergillus nidulans*					JGI	2011/06
Eurotiomycetes	*Aspergillus oryzae*					JGI	2011/06

Eurotiomycetes	*Arthroderma otae*					The Broad Institute	2011/06
Pezizomycotina	*Aspergillus terreus*					JGI	2011/06
Pezizomycotina	*Botryotinia fuckeliana*					JGI	2011/06
Eurotiomycetes	*Ajellomyces dermatitidis*					The Broad Institute	2011/06
Sordariomycetes	*Chaetomium globosum*					JGI	2011/06
Ascomycetes	*Cochliobolus heterostrophus*					JGI	2011/06
Sordariomycetes	*Colletotrichum higginsianum*					The Broad Institute	2011/06
Eurotiomycetes	*Coccidioides immitis*					The Broad Institute	2011/06
Eurotiomycetes	*Coccidioides posadasii*					The Broad Institute	2011/06
Sordariomycetes	*Cryphonectria parasitica*					JGI	2011/06
Sordariomycetes	*Fusarium oxysporum*					The Broad Institute	2011/06
Sordariomycetes	*Gibberella moniliformis*					The Broad Institute	2011/06
Sordariomycetes	*Glomerella graminicola*					The Broad Institute	2011/06
Sordariomycetes	*Gibberella zeae*					JGI	2011/06
Dothideomycetes	*Mycosphaerella fijiensis*					JGI	2011/06
Dothideomycetes	*Mycosphaerella graminicola*					JGI	2011/06
Eurotiomycetes	*Microsporum gypseum*					The Broad Institute	2011/06
Dothideomycetes	*Mycosphaerella pini*					JGI	2011/06
Sordariomycetes	*Magnaporthe oryzae*					JGI	2011/06
Dothideomycetes	*Mycosphaerella populorum*					JGI	2011/06
Sordariomycetes	*Myceliophthora thermophila*					JGI	2011/06
Ascomycetes	*Neurospora crassa*					JGI	2011/06
Ascomycetes	*Neurospora discreta*					JGI	2011/06
Eurotiomycetes	*Neosartorya fischeri*					JGI	2011/06
Sordariomycetes	*Nectria haematococca*					JGI	2011/06
Ascomycetes	*Neurospora tetrasperma*					JGI	2011/06
Sordariomycetes	*Podospora anserina*					JGI	2011/06
Eurotiomycetes	*Paracoccidioides brasiliensis*					The Broad Institute	2011/06
Eurotiomycetes	*Penicillium chrysogenum*					JGI	2011/06
Dothideomycetes	*Phaeosphaeria nodorum*					JGI	2011/06
Dothideomycetes	*Pyrenophora tritici-repentis*					The Broad Institute	2011/06

Leotiomycetes	*Sclerotinia sclerotiorum*					JGI	2011/06
Sordariomycetes	*Trichoderma atroviride*					JGI	2011/06
Eurotiomycetes	*Trichophyton equinum*					The Broad Institute	2011/06
Pezizomycetes	*Tuber melanosporum*					KEGG	2011/06
Eurotiomycetes	*Trichophyton rubrum*					The Broad Institute	2011/06
Sordariomycetes	*Hypocrea jecorina*					JGI	2011/06
Eurotiomycetes	*Trichophyton tonsurans*					The Broad Institute	2011/06
Sordariomycetes	*Thielavia terrestris*					JGI	2011/06
Eurotiomycetes	*Trichophyton verrucosum*					The Broad Institute	2011/06
Sordariomycetes	*Hypocrea virens*					JGI	2011/06
Eurotiomycetes	*Uncinocarpus reesii*					The Broad Institute	2011/06
Sordariomycetes	*Verticillium albo-atrum*					The Broad Institute	2011/06
Sordariomycetes	*Verticillium dahliae*					The Broad Institute	2011/06
Saccharomycetes	*Ashbya gossypii*					YGOB	2011/06
Saccharomycetes	*Candida albicans*					The Broad Institute	2011/06
Saccharomycetes	*Candida dubliniensis*					KEGG	2011/06
Saccharomycetes	*Candida glabrata*					Genolevures	2011/06
Saccharomycetes	*Clavispora lusitaniae*					The Broad Institute	2011/06
Saccharomycetes	*Candida parapsilosis*					The Broad Institute	2011/06
Saccharomycetes	*Candida tropicalis*					The Broad Institute	2011/06
Saccharomycetes	*Debaryomyces hansenii*					Genolevures	2011/06
Saccharomycetes	*Kluyveromyces lactis*					Genolevures	2011/06
Saccharomycetes	*Vanderwaltozyma polyspora*					YGOB	2011/06
Saccharomycetes	*Lachancea waltii*					Duke	2011/06
Saccharomycetes	*Lodderomyces elongisporus*					JGI	2011/06
Saccharomycetes	*Lachancea thermotolerans*					JGI	2011/06
Saccharomycetes	*Meyerozyma guilliermondii*					JGI	2011/06
Saccharomycetes	*Pichia pastoris*					JGI	2011/06
Saccharomycetes	*Scheffersomyces stipitis*					JGI	2011/06
Saccharomycetes	*Saccharomyces bayanus*					YGOB	2011/06
Saccharomycetes	*Naumovia castellii*					YGOB	2011/06

Saccharomycetes	*Saccharomyces cerevisiae*					SGD	2011/06
Saccharomycetes	*Lachancea kluyveri*					Genolevures	2011/06
Saccharomycetes	*Saccharomyces kudriavzevii*					The Hyphal Tip	2011/06
Saccharomycetes	*Saccharomyces mikatae*					The Hyphal Tip	2011/06
Saccharomycetes	*Saccharomyces paradoxus*					The Hyphal Tip	2011/06
Saccharomycetes	*Yarrowia lipolytica*					Genolevures	2011/06
Saccharomycetes	*Zygosaccharomyces rouxii*					JGI	2011/06
Schizosaccharomycetes	*Schizosaccharomyces cryophilus*					The Broad Institute	2011/06
Schizosaccharomycetes	*Schizosaccharomyces japonicus*					The Broad Institute	2011/06
Schizosaccharomycetes	*Schizosaccharomyces octosporus*					The Broad Institute	2011/06
Schizosaccharomycetes	*Schizosaccharomyces pombe*					The Broad Institute	2011/06

Table 3.

List of 28 completely sequenced Basidiomycota genomes. Columns indicate, in this order, the taxonomic group, the species, the source from where proteomes were obtained, and the date the data was acquired. Gray boxes indicate which species were used as queries for the orthology searches.

Taxonomic Group	Scientific Name	Source	As of
Agaricomycotina	*Tremella mesenterica*	JGI	2011/06
Agaricomycotina	*Phanerochaete chrysosporium*	JGI	2011/06
Agaricomycotina	*Trametes versicolor*	JGI	2011/06
Agaricomycotina	*Schizophyllum commune*	JGI	2011/06
Agaricomycotina	*Agaricus bisporus*	JGI	2011/06
Agaricomycotina	*Coprinopsis cinerea*	JGI	2011/06
Agaricomycotina	*Laccaria bicolor*	JGI	2011/06
Agaricomycotina	*Fomitopsis pinicola*	JGI	2011/06
Agaricomycotina	*Stereum hirsutum*	JGI	2011/06
Agaricomycotina	*Ceriporiopsis subvermispora*	JGI	2012/04
Agaricomycotina	*Coniophora puteana*	JGI	2011/06
Agaricomycotina	*Wolfiporia cocos*	JGI	2011/06
Agaricomycotina	*Gloeophyllum trabeum*	JGI	2011/06

Agaricomycotina	*Dichomitus squalens*	JGI	2011/06
Agaricomycotina	*Punctularia strigosozonata*	JGI	2011/06
Agaricomycotina	*Fomitiporia mediterranea*	JGI	2011/06
Agaricomycotina	*Cryptococcus neoformans*	JGI	2011/06
Agaricomycotina	*Cryptococcus gattii*	UniProt	2012/04
Pucciniomycotina	*Puccinia graminis*	JGI	2011/06
Pucciniomycotina	*Rhodotorula graminis*	JGI	2011/06
Pucciniomycotina	*Melampsora larici-populina*	JGI	2011/06
Pucciniomycotina	*Puccinia triticina*	The Broad Institute	2011/06
Pucciniomycotina	*Mixia osmundae*	JGI	2012/04
Ustilaginomycotina	*Malassezia globosa*	JGI	2011/06
Ustilaginomycotina	*Ustilago maydis*	JGI	2012/04
Ustilaginomycotina	*Sporisorium reilianum*	UniProt	2012/04
Ustilaginomycotina	*Ustilago hordei*	UniProt	*2013/02*
Wallemiomycetes	*Wallemia sebi*	JGI	2011/06

Table 4.

Results obtained when *one-genome-at-the-time* test was applied to the 19 cyanobacterial genomes belonging to the Testing set. Columns indicate, in this order, the scientific name of the studied species; the number of selected markers found in each species (out of 6); the number of all marker genes found (out of 203 initial set); the Robinson & Foulds distance (RF) (18), which measures topological differences, between the tree inferred using the selected markers and all available marker genes when each species is considered; percentage of wrong splits as a normalized measure of topological differences between the trees inferred using the selected markers or all available markers for a given species.

Scientific Name	Selected Markers (6)	All Markers (203)	R&F distance	% Wrong splits
Cyanobacterium UCYN-A	5	162	0	0%
Oscillatoria sp. PCC 6506	5	189	0	0%
Cyanothece sp. CCY0110	6	198	0	0%
Gloeothece sp. PCC 6909/1	6	198	0	0%

Prochlorococcus marinus str. MIT9202	6	196	0	0%
Raphidiopsis brookii D9	6	198	0	0%
Cyanothece sp. (str. PCC 7822)	6	200	0	0%
Cylindrospermopsis raciborskii CS-505	6	200	0	0%
Synechococcus sp. BL107	6	202	0	0%
Synechococcus sp. WH 8109	6	203	0	0%
Coleofasciculus chthonoplastes PCC7420	6	197	2	2.38 %
Cyanobium sp. PCC 7001	6	197	2	2.38 %
Synechococcus sp. CB0205	6	200	2	2.38 %
Synechococcus sp. CB0101	6	201	2	2.38 %
Synechococcus sp. RS9916	6	201	2	2.38 %
Synechococcus sp. RS9917	6	202	2	2.38 %
Prochlorococcus sp. UH18301	6	201	2	4.76 %
Nostoc azollae (strain 0708)	6	199	4	4.76 %

Table 5.

Results for the concatenation of traditionally used marker genes in Cyanobacteria (22, 23). Only coding-protein genes were considered for the comparison in order to use the same methodology regarding the BLAST search, multiple sequence alignment and phylogenetic tree reconstruction. Since *nifD* was absent, present in multiple copies or with a low coverage for many species, we constructed a second set of marker genes without it. Comparisons in terms of topological differences, were performed against the different reference species trees for the training, testing and all cyanobacterial species.

**Combination of marker genes**	**Training (43 species)**		**Testing (19 species)**		**All (62 species)**	
	(1)	(2)	(1)	(2)	(1)	(2)
gyrB + rpoC1 + rpoD1 + nifD	12	14.63%	2	5.88%	32	26.67%
gyrB + rpoC1 + rpoD1	14	17.07%	4	11.76%	24	20 %

(1): R&F distance

(2): % Wrong splits

Table 6.

Results for the concatenation of all possible combinations of three and four genes of the final set of marker genes for cyanobacteria. The logic behind this comparison is to compare the performance of the combination of markers with similar size against the two possible combinations (of 3 and 4 genes) of the traditional coding-protein markers. Comparisons, in terms of topological differences, were performed against the different reference species trees for the training, testing and all cyanobacterial species.

**Dataset**	**Number of genes**	**Training (43 species)**		**Testing (19 species)**		**All (62 species)**	
		(1)	(2)	(1)	(2)	(1)	(2)
01	3	14	32.56 %	0	0 %	18	29.03 %
02	3	8	18.60 %	2	10.53 %	10	16.13 %
03	3	8	18.6 %	2	10.53 %	10	16.13 %
04	3	12	27.91 %	2	10.53 %	12	19.35 %
05	3	10	23.26 %	0	0 %	20	32.26 %
06	3	8	18.60 %	2	10.53 %	22	35.48 %
07	3	8	18.60 %	4	21.05 %	18	29.03 %
08	3	10	23.26 %	2	10.53 %	24	38.71 %
09	3	10	23.26 %	0	0 %	26	41.94 %
10	3	6	13.95 %	2	10.53 %	16	25.81 %
11	3	6	13.95 %	4	21.05 %	22	35.48 %
12	3	12	27.91 %	2	10.53 %	20	32.26 %
13	3	4	9.302 %	4	21.05 %	14	22.58 %
14	3	10	23.26 %	0	0 %	18	29.03 %
15	3	4	09.30 %	0	0 %	10	16.13 %
16	3	10	23.26 %	0	0 %	16	25.81 %
17	3	2	04.65 %	0	0 %	14	22.58 %
18	3	8	18.60 %	0	0 %	22	35.48 %
19	3	6	13.95 %	0	0 %	16	25.81 %
20	3	8	18.60 %	2	10.53 %	14	22.58 %
21	3	8	18.60 %	2	10.53 %	12	19.35 %
22	3	4	09.30 %	0	0 %	8	12.90 %
23	3	6	13.95 %	0	0 %	14	22.58 %
24	3	4	09.30 %	0	0 %	16	25.81 %
25	3	2	04.65 %	0	0 %	16	25.81 %
26	3	10	23.26 %	4	21.05 %	16	25.81 %
27	3	12	27.91 %	2	10.53 %	26	41.94 %
28	3	10	23.26 %	0	0 %	18	29.03 %
29	3	6	13.95 %	10	52.63 %	18	29.03 %

30	3	8	18.60 %	0	0 %	16	25.81 %
31	3	4	09.30 %	0	0 %	22	35.48 %
32	3	10	23.26 %	4	21.05 %	18	29.03 %
33	3	8	18.60 %	2	10.53 %	12	19.35 %
34	3	10	23.26 %	2	10.53 %	16	25.81 %
35	3	6	13.95 %	2	10.53 %	14	22.58 %
36	4	0	0 %	0	0 %	12	19.35 %
37	4	0	0 %	0	0 %	10	16.13 %
38	4	8	18.60 %	0	0 %	14	22.58 %
39	4	4	09.30 %	0	0 %	14	22.58 %
40	4	0	0 %	2	10.53 %	14	22.58 %
41	4	0	0 %	2	10.53 %	12	19.35 %
42	4	4	09.30 %	2	10.53 %	14	22.58 %
43	4	4	09.30 %	2	10.53 %	12	19.35 %
44	4	8	18.6 %	0	0 %	8	12.90 %
45	4	2	04.65 %	0	0 %	8	12.90 %
46	4	0	0 %	0	0 %	18	29.03 %
47	4	4	09.30 %	2	10.53 %	16	25.81 %
48	4	10	23.26 %	0	0 %	14	22.58 %
49	4	6	13.95 %	2	10.53 %	18	29.03 %
50	4	8	18.60 %	0	0 %	16	25.81 %
51	4	2	04.65 %	0	0 %	14	22.58 %
52	4	0	0 %	4	21.05 %	14	22.58 %
53	4	10	23.26 %	0	0 %	14	22.58 %
54	4	6	13.95 %	2	10.53 %	14	22.58 %
55	4	6	13.95 %	0	0 %	12	19.35 %
56	4	0	0 %	0	0 %	14	22.58 %
57	4	10	23.26 %	0	0 %	20	32.26 %
58	4	2	04.65 %	0	0 %	12	19.35 %
59	4	8	18.60 %	0	0 %	16	25.81 %
60	4	2	04.65 %	0	0 %	18	29.03 %
61	4	0	0 %	0	0 %	14	22.58 %
62	4	6	13.95 %	0	0 %	16	25.81 %
63	4	2	04.65 %	0	0 %	8	12.90 %
64	4	4	09.30 %	0	0 %	10	16.13 %
65	4	2	04.65 %	0	0 %	10	16.13 %
66	4	10	23.26 %	4	21.05 %	24	38.71 %
67	4	6	13.95 %	4	21.05 %	12	19.35 %
68	4	8	18.60 %	0	0 %	12	19.35 %
69	4	6	13.95 %	0	0 %	16	25.81 %
70	4	6	13.95 %	2	10.53 %	14	22.58 %

(1): R&F distance

(2): % Wrong splits

Table 7.

Results obtained when *one-genome at the time* test was applied to the 28 fungal genomes from Ascomycota belonging to the testing set. In this order, the scientific name of the studied species, the number of selected markers found in each species (out of 4); the number of all marker genes found (out of 169 initial set); the Robinson & Foulds distance (RF) (18), which measures topological differences, between the tree inferred using the selected markers and all available marker genes when each species is considered; percentage of wrong splits as a normalized measure of topological differences between the trees inferred using the selected markers or all available markers for a given species.

Scientific Name	Selected Markers (4)	All Markers (169)	R&F distance	% Wrong splits
Saccharomyces kudriavzevii	3	132	0	0 %
Schizosaccharomyces octosporus	3	146	0	0 %
Saccharomyces bayanus	4	135	0	0 %
Sclerotinia sclerotiorum	4	143	0	0 %
Gibberella moniliformis	4	143	0	0 %
Uncinocarpus reesii	4	145	0	0 %
Candida tropicalis	4	146	0	0 %
Verticillium dahliae	4	147	0	0 %
Trichoderma atroviride	4	147	0	0 %
Mycosphaerella graminicola	4	147	0	0 %
Ajellomyces dermatitidis	4	147	0	0 %
Gibberella zeae	4	148	0	0 %
Trichophyton verrucosum	4	149	0	0 %
Mycosphaerella pini	4	150	0	0 %
Pyrenophora tritici-repentis	3	147	2	1.85 %
Tuber melanosporum	4	139	2	1.85 %
Alternaria brassicicola	4	140	2	1.85 %
Saccharomyces paradoxus	4	145	2	1.85 %

Microsporum gypseum	4	145	2	1.85 %
Candida glabrata	4	147	2	1.85 %
Scheffersomyces stipitis	4	148	2	1.85 %
Thielavia terrestris	4	151	2	1.85 %
Magnaporthe oryzae	4	153	2	1.85 %
Aspergillus oryzae	4	134	4	3.70 %
Aspergillus flavus	4	136	4	3.70 %
Zygosaccharomyces rouxii	4	150	4	3.70 %
Kluyveromyces lactis	4	150	4	3.70 %
Penicillium chrysogenum	4	140	6	5.56 %

Table 8.

Results for the concatenation of marker genes for the fungal Species Tree. On this comparison only coding-protein genes were considered in order to use the same methodology for the BLAST search, Multiple Sequence Alignment and phylogenetic tree reconstruction as for our set of marker genes. Two datasets were used on this analysis, one containing 4 broadly used markers and the other one containing the two markers proposed by (3). Tree topologies comparisons were performed for the training, testing and all sets of species considered on this study.

**Combination of marker genes**	**Training (55 species)**		**Testing (28 species)**		**All (83 species)**	
	(1)	(2)	(1)	(2)	(1)	(2)
tef1 + tub2 + tsr1 + cdc47p	12	11.32%	6	11.54%	20	12.35%
trs1 + cdc47p	8	7.55%	6	11.54%	24	14.81%

(1): R&F distance

(2): % Wrong splits

Table 9.

Sets of selected marker genes for the two additional rounds performed on Ascomycota. Protein information was retrieved from UNIPROT using either *Saccharomyces cerevisiae* (Ascomycota - round 2) or *Candida glabrata* (Ascomycota - round 3).

Round	**Uniprot Id**	**Length (AA)**	**Annotation**
2	**P17883**	1432	Superkiller protein 3
2	**P32855**	1065	Exocyst complex component SEC8
2	**Q02939**	513	RNA polymerase II transcription factor B subunit 2
2	**Q12059**	462	NEDD8-activating enzyme E1 regulatory subunit
2	**Q03290**	321	RNA polymerase II transcription factor B subunit 3
3	**Q6FLD0**	963	mRNA transport regulator MTR10
3	**Q6FSR7**	889	phosphatidylinosit ol 3-kinase
3	**Q6FNC4**	634	Translation initiation factor eIF-2B subunit delta
3	**Q6FP41**	504	RNA polymerase II transcription factor B subunit 2
3	**Q6FQP1**	296	ATP synthase subunit gamma

Table 10.

Pearson's correlation for alternative distances to the Robinson and Foulds distance (RF) (18). Tree certainty (TC) (6), Nodal distance (ND) (35), K-tree score (KT) (20) and likelihood ratio (LK) were computed for all datasets and its corresponding correlation to the RF used along this study.

**Dataset / Distaces**	**TC**		**ND**		**KT**		**LK**	
	**1**	**2**	**1**	**2**	**1**	**2**	**1**	**2**
**Cyanobacteria**	-0.9273	9.56e-088	0.7503	5.62e-038	0.4787	5.02e-013	0.1199	8.83e-002
**Ascomycota - Round 1**	-0.8973	3.46e-061	0.8108	1.10e-040	0.5213	3.68e-013	0.3307	1.12e-005
**Ascomycota - Round 2**	-0.9135	1.41e-074	0.8262	3.17e-048	0.5518	2.28e-016	0.3552	5.70e-007
**Ascomycota - Round 3**	-0.9407	6.90e-063	0.8339	2.35e-035	0.5477	1.07e-011	0.2734	1.51e-003
**Ascomycota - Round 4**	-0.9313	1.96e-109	0.8825	3.00e-082	0.6256	3.06e-028	0.4650	1.19e-014
**Basidiomycota**	-0.9225	1.38e-236	0.8253	7.76e-143	0.4521	5.22e-030	0.5097	6.05e-039

(1) Pearson's r

(2) p-value

2. Supplementary figures.

Figure S1.

Percentage of wrong splits against the reference tree, at each step of the progressive concatenation of 201 gene-sets . Progressive concatenation was performed according to the distance of individual gene-sets to the reference topology. As indicated on the figure, at least 35 genes are needed to recover the same topology as the reference species tree. The reference species tree was reconstructed after concatenating 203 sets of single-copy widespread genes across 43 cyanobacterial species from the training set.

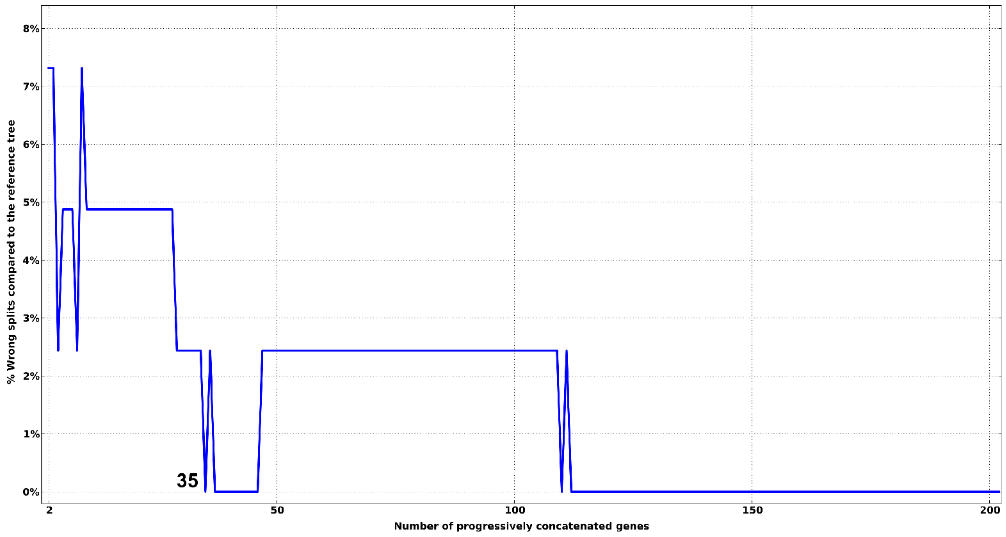

Figure S2.

Percentage of wrong splits of the progressive concatenation of 167 gene-sets compared to the reference species tree. Progressive concatenation was performed following the order of the distance of individual markers to the reference topology using different alternative measurements: Robinson and Foulds distance (blue line), Tree Certainty (red line), K-tree score (green line) and Likelihood ratio (cyan line). On the figure is marked how many genes are needed, for each ranking approximation, to concatenate for recovering the reference tree topology. The reference species tree was reconstructed after concatenating 169 sets of single-copy widespread genes across 55 ascomycotal fungal species from the training set.

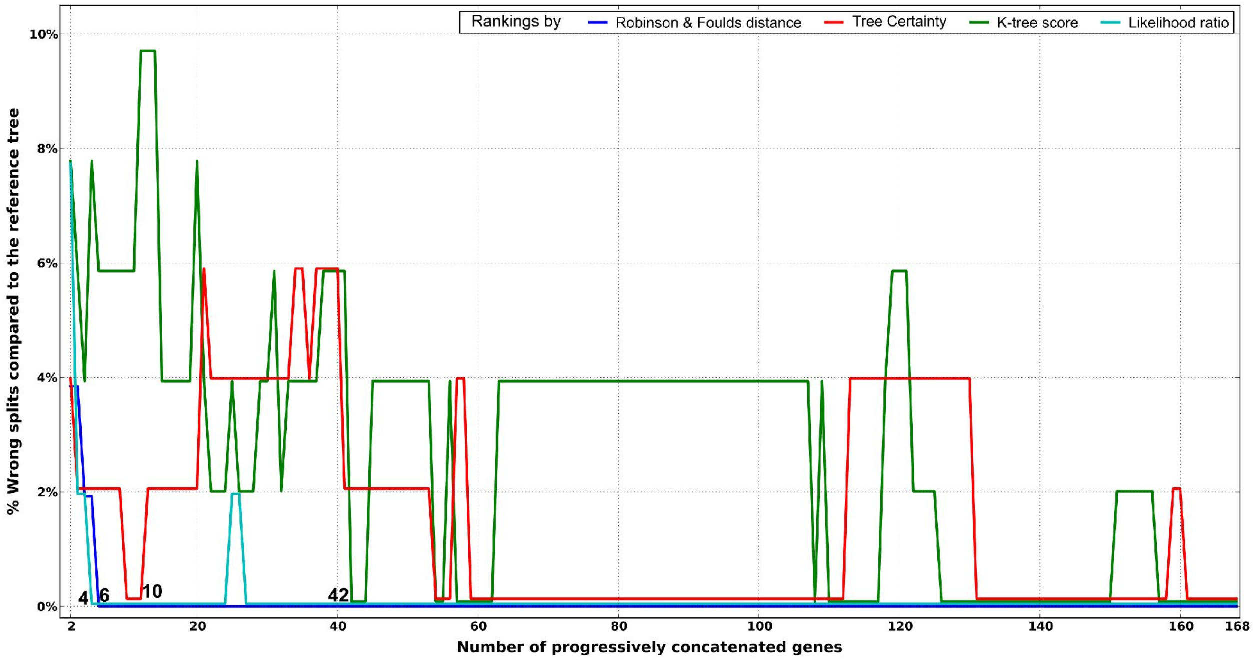

Figure S3.

Percentage of wrong splits for 615 concatenated gene-sets. Gene-sets were constructed as random combinations of the initial marker genes sets (6 gene-sets). The combination yielding the same topology as the reference which was used for downstream analyses is marked on the figure with stars.

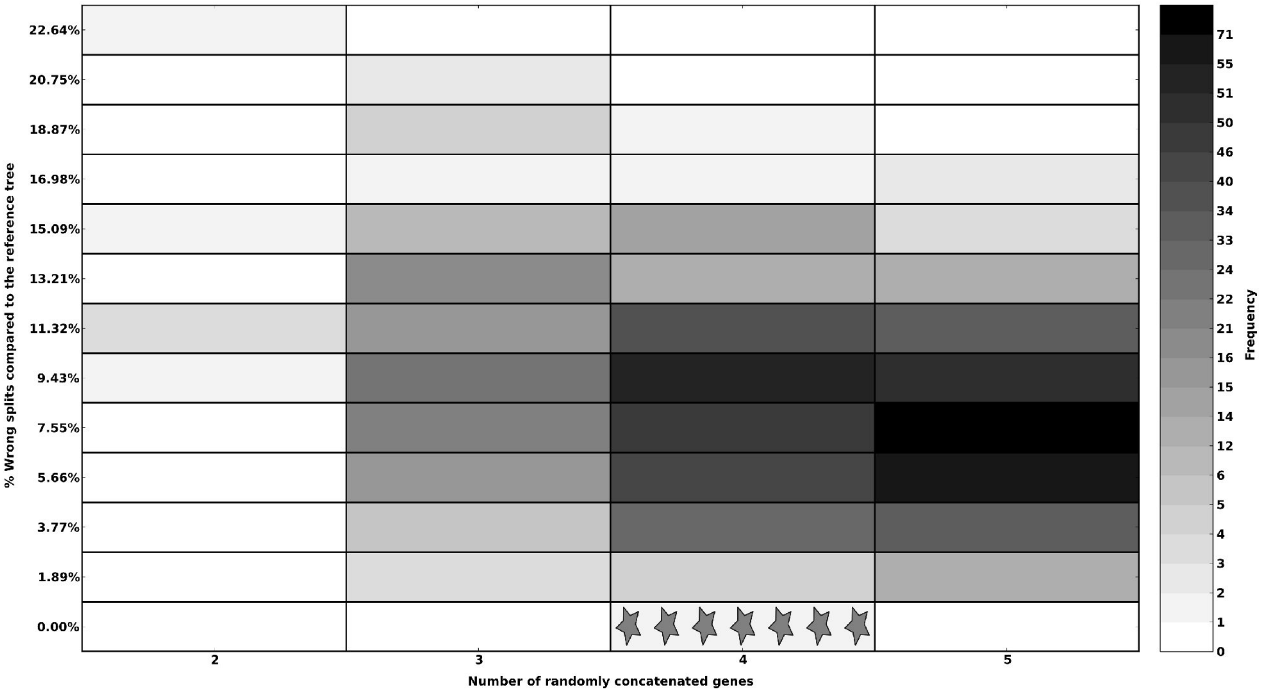

Figure 4.

Percentage of wrong splits, as compared with the reference tree, for 1,000 different combinations of 4 marker genes selected among the available ones for the Ascomycota - Round 1 experiment. The idea behind this experiment is to measure how often a combination of 4 marker genes, the size of the one selected in our experiment, are found. Considering the average (8.9440) and the standard deviation (+/- 3.7538) of the distribution, having a value of 0% (same reference topology) is further than (avg + 2std) which covers the 95% of the expected values.

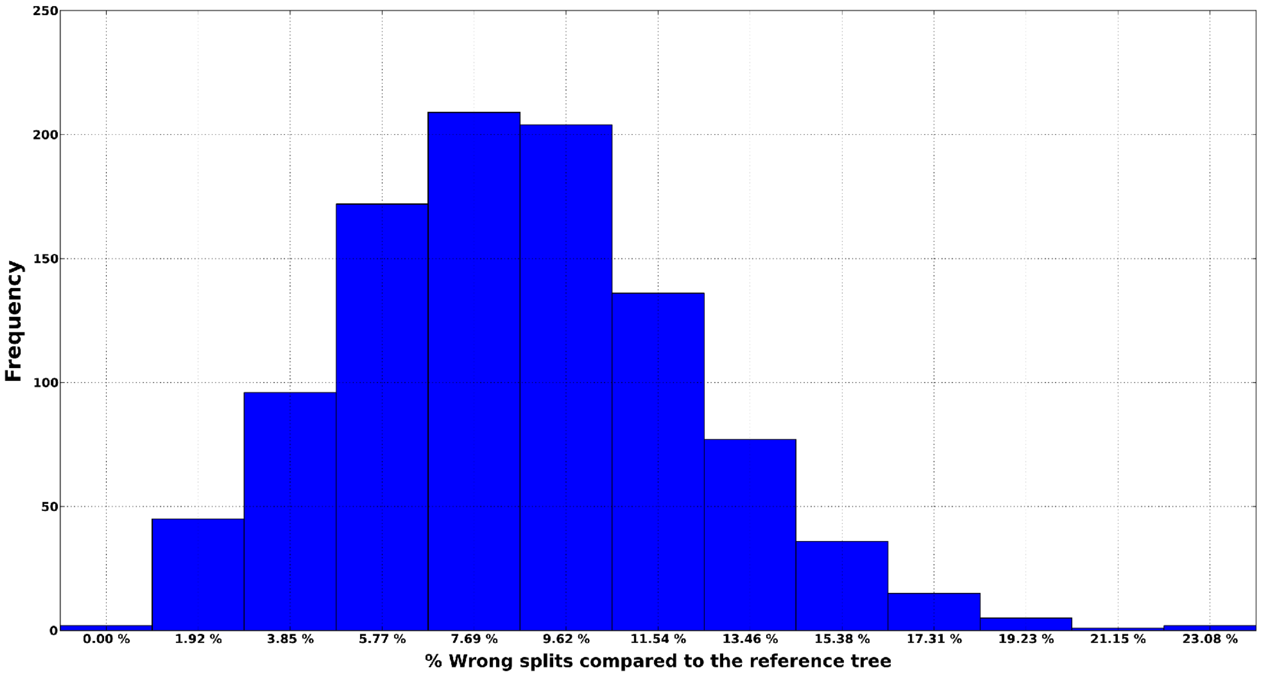

Figure S5.

Basidiomycota fungal species tree comprising 28 species used as an additional validation step for the selection of 4 gene markers set on Ascomycota. The same tree topology was recovered using either the concatenation of the 4 gene markers set or the concatenation of 313 single-copy widespread genes present in all species. Gray boxes indicate which species were used as query to perform the BLAST search of orthologous genes.

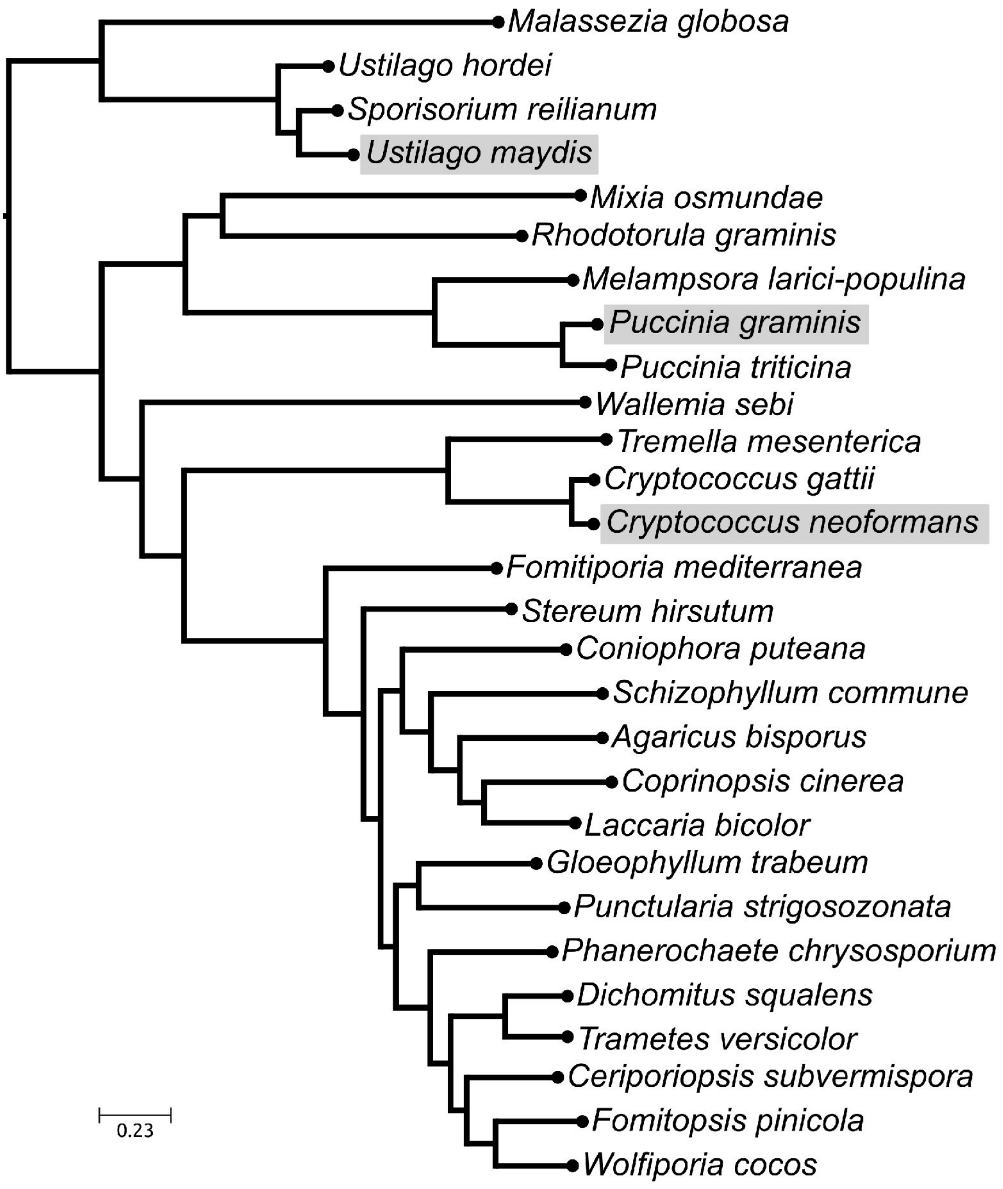

3. Pipeline description in pseudo-code.

1) Divide the initial set of species into non-overlapping training (T-set) and validation (V-set) sets. Size of each set is optional, by default 2:1 (T:V) size ratio is used.

2) Identify marker genes as genes with single copy orthologs in 100% (by default) of the species in the T-set. 100% can be optionally lowered.

3) For each marker gene identified in step 2, reconstruct a multiple sequence alignment (MSA).

4) Generate the reference topology for the T-set by concatenating all alignments generated in step 3.

5) Rank individual markers by their distance to the reference topology using any desired metric (e.g. Robinson and Foulds distance, Tree Certainty, Likelihood ratio)

6) Generate, progressively, concatenated alignments from the most similar to the most dissimilar markers up to reaching a given threshold. By default, the threshold is set to recover exactly the same reference topology (distance = 0). Even when the threshold is reached, the process can be extended to explore all progressively concatenated markers.

7) The set of concatenated markers for which, for the first time, a given threshold is reached constitutes the initial markers set. Depending on the size of such set, it could be tested directly (go to step 9) or start random concatenation of markers to reduce the size of this set.

8) In order to reduce the initial markers set size, smaller sets can be randomly generated using either only markers from the initial set or from the whole set of markers. The number of randomly concatenated sets can be set to a given number, e.g. 100 or 1000, or explored completely when the number of combinations is affordable. For instance, there are 1,024 possible combinations of sizes 2 to 9 for an initial marker set of 10 genes, conversely, for an initial set of 20 markers there are 1,048,576 possible combinations.

9) Identify which previously identified markers are on the V-set of species. Markers are required to be single copy but not widespread across all species.

10) Reference topologies are reconstructed following two approximations:

> 10A) a reference tree is reconstructed only for species in the V-set by concatenating the markers found in step 9.
>
> 10B) a tree for each individual species in the validation set is reconstructed which includes species in the training set plus each individual species.

11) Following a similar strategy to step 10, trees using only markers identified in steps 7 or 8 are reconstructed. Then, tree topologies recovered for the selected markers are compared against the one rendered using all markers found in V-set. In this way, the phylogenetic signal carried by the set of markers is evaluated. It is also evaluated the presence or not of individual markers on the newly set of species.

12) Depending on the results in the previous step, a set of markers can be proposed as the outcome of the experiment, if satisfactory. If new sets of markers are needed, go to step 8 exploring more random combination or increasing the upper limit of the marker genes set size.

13) A final tree including all species used in the study is reconstructed using either all available marker or only the selected ones. Comparison of both topologies gives an idea about the potential of the selected set of marker genes for resolving large species trees.
